# Nanoarchitectonics-based model membrane platforms for probing membrane-disruptive interactions of odd-chain antimicrobial lipids

**DOI:** 10.1186/s40580-022-00339-1

**Published:** 2022-11-01

**Authors:** Bo Kyeong Yoon, Sue Woon Tan, Jia Ying Brenda Tan, Joshua A. Jackman, Nam-Joon Cho

**Affiliations:** 1 School of Materials Science and Engineering, Nanyang Technological University, Singapore, 639798 Singapore; 2 School of Chemical Engineering and Translational Nanobioscience Research Center, Sungkyunkwan University, Suwon, 16419 Republic of Korea; 3 School of Healthcare and Biomedical Engineering, Chonnam National University, Yeosu, 59626 Republic of Korea

**Keywords:** Antimicrobial lipid, Supported lipid bilayer, Tethered lipid bilayer, Quartz crystal microbalance-dissipation, Electrochemical impedance spectroscopy

## Abstract

The use of nanoscience tools to investigate how antimicrobial lipids disrupt phospholipid membranes has greatly advanced molecular-level biophysical understanding and opened the door to new application possibilities. Until now, relevant studies have focused on even-chain antimicrobial lipids while there remains an outstanding need to investigate the membrane-disruptive properties of odd-chain antimicrobial lipids that are known to be highly biologically active. Herein, using the quartz crystal microbalance-dissipation (QCM-D) and electrochemical impedance spectroscopy (EIS) techniques, we investigated how an 11-carbon, saturated fatty acid and its corresponding monoglyceride—termed undecanoic acid and monoundecanoin, respectively—disrupt membrane-mimicking phospholipid bilayers with different nanoarchitectures. QCM-D tracking revealed that undecanoic acid and monoundecanoin caused membrane tubulation and budding from supported lipid bilayers, respectively, and were only active above their experimentally determined critical micelle concentration (CMC) values. Monoundecanoin was more potent due to a lower CMC and electrochemical impedance spectroscopy (EIS) characterization demonstrated that monoundecanoin caused irreversible membrane disruption of a tethered lipid bilayer platform at sufficiently high compound concentrations, whereas undecanoic acid only induced transient membrane disruption. This integrated biophysical approach also led us to identify that the tested 11-carbon antimicrobial lipids cause more extensive membrane disruption than their respective 12-carbon analogues at 2 × CMC, which suggests that they could be promising molecular components within next-generation antimicrobial nanomedicine strategies.

## Introduction

Antimicrobial lipids such as fatty acids and monoglycerides are single-chain lipid amphiphiles that can exhibit antimicrobial activity against a wide range of bacteria, yeast, fungi, enveloped viruses, protozoa, and algae [1–3]. Owing to their amphipathic nature, fatty acids and monoglycerides are considered membrane-disruptive agents and are understood to act as mild surfactants that destabilize pathogenic membranes [4]. In light of the forthcoming “post-antibiotic era” [5–9], the demonstrated effectiveness of these antimicrobial lipids against antibiotic-resistant bacterial strains such as methicillin-resistant *S. aureus* [10–12] has further heightened interest in understanding their membrane-related antimicrobial activities and corresponding membrane biophysics.

Pioneering works by Kabara and colleagues examined the potency and scope of antimicrobial activity of saturated fatty acids and monoglycerides with different chain lengths and identified that medium-chain (typically between 6 and 12 carbons long) saturated fatty acids and their 1-monoglyceride derivatives, notably the 10- and 12-carbon long ones, have the highest antimicrobial potency [13–15]. Of note, 12-carbon lauric acid (LA) was the most active saturated fatty acid to inhibit Gram-positive bacteria, while glycerol monolaurate (GML), which is the monoglyceride derivative of LA, had even greater inhibitory potency [14, 16]. Additionally, 10-carbon capric acid and its monoglyceride derivative, monocaprin, were found to have greater inhibitory activity against Gram-negative bacteria [13, 17, 18]. To date, studies on fatty acids and monoglycerides have mainly focused on how their antibacterial activity spectra can be related to structural variations such as carbon chain length and degree of saturation [5, 18–21]. In addition, it has been shown that there is a high barrier for pathogens such as bacteria to evolve and acquire resistance to antimicrobial lipids [5, 22, 23]. Such efforts have provided empirical insight into structure-activity relationships [23–26], while there has also been interest into building a physicochemical understanding of the underpinning molecular-level interactions between antimicrobial lipids and phospholipid membranes [27].

**Fig. 1:**
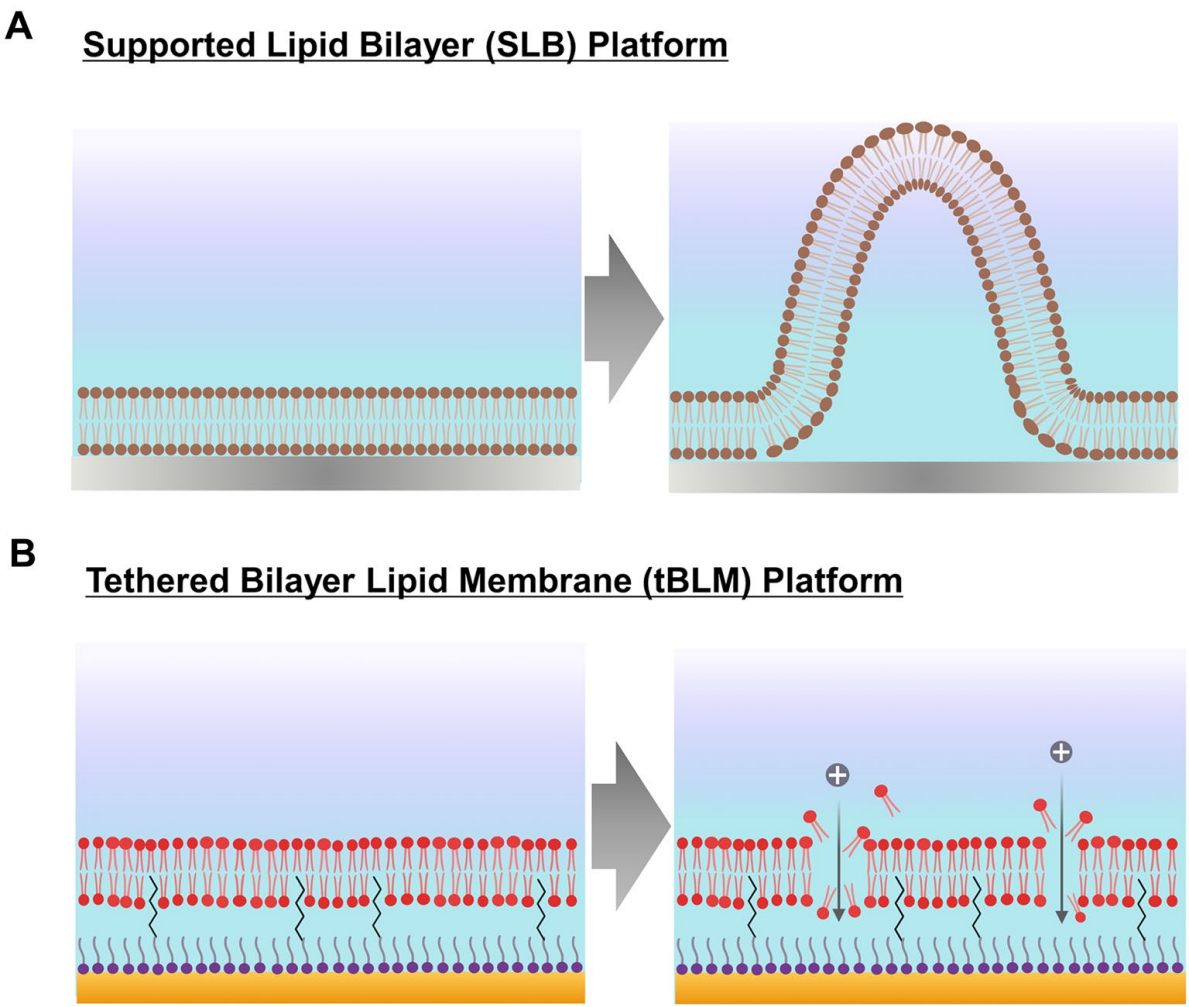
Schematic illustration of nanoarchitectonics-based model membrane platforms for characterizing membrane-disruptive interactions of antimicrobial lipids. **A** Supported lipid bilayer (SLB) platform enables tracking membrane morphological changes when used in conjunction with the quartz crystal microbalance-dissipation (QCM-D) technique. **B** Tethered bilayer lipid membrane (tBLM) platform enables tracking ionic permeability changes when used in conjunction with the electrochemical impedance spectroscopy (EIS) technique

Within this scope, recent attention has been placed on utilizing biophysical measurement strategies to track the real-time interactions between antimicrobial lipids and engineered, membrane-mimicking phospholipid membranes, especially when combined with nanoarchitectonics-based sensing platforms (Fig. 1) [28]. Among the different options, supported lipid bilayers (SLBs) have proven especially useful to track interactions between antimicrobial lipids and phospholipid membranes because they can be studied using a wide range of surface-sensitive measurement techniques [29–36]. Using the quartz crystal microbalance-dissipation (QCM-D) and time-lapse fluorescence microscopy techniques, Yoon et al. found that the addition of 10-carbon capric acid, at concentrations above its critical micelle concentration (CMC), to a phospholipid-based SLB induced morphological changes that included the formation of protruding tubules [37]. At lower concentrations below the CMC, capric acid did not disrupt the SLB. By contrast, its monoglyceride equivalent, monocaprin, caused the formation of elongated tubules and membrane buds below and above its CMC, respectively, and was generally more potent than capric acid. Another study on 12-carbon antimicrobial lipids noted that, above their corresponding CMC values, the interaction of LA and a related anionic surfactant, sodium dodecyl sulfate (SDS), with SLB platforms caused the formation of protruding tubules that led to incomplete disruption or complete solubilization, respectively, while GML triggered membrane budding [38]. These studies established that differences in chemical structure, even among single-chain antimicrobial lipids with the same carbon length, can result in distinct interactions with phospholipid membranes, which was rationalized by taking into account factors such as headgroup charge and resulting effects on micellar aggregation and inter-leaflet bilayer translocation [39].

Other label-free biosensing techniques such as localized surface plasmon resonance (LSPR) have provided additional insights into how LA, SDS, and GML disrupt SLBs in terms of three-dimensional membrane shape transformations [40]. In addition to the SLB platform, there have also been efforts to understand how antimicrobial lipids disrupt the ionic permeability of tethered lipid bilayer membranes (tBLM) by using the electrochemical impedance spectroscopy (EIS) technique [48]. Hence, a variety of label-free biosensing techniques have been established to study membrane-disruptive interactions, whereby options like QCM-D and LSPR mainly focus on tracking three-dimensional membrane morphological changes while EIS tracks changes in membrane conductance and capacitance. To date, these two approaches have been utilized independently in separate studies while combining such approaches would be useful to comprehensively evaluate antimicrobial lipids that have not yet been characterized using biophysical approaches. This gap is especially true for odd-chain saturated fatty acids such as the 11-carbon long, saturated undecanoic acid, which is known to have high antifungal activity compared to other medium-chain fatty acids [41, 42], and its corresponding monoglyceride, monoundecanoin, which is also regarded as having high antimicrobial activity [43]. Antimicrobial lipids built from even-chain fatty acid building blocks are more common in nature, especially in mammals, and hence more widely studied while biosynthetic pathways exist to produce odd-chain fatty acids and understanding their molecular-level interaction behavior can potentially lead to new application possibilities.

Herein, using the QCM-D and EIS techniques, we investigated the membrane-disruptive interactions of undecanoic acid and monoundecanoin in terms of potency and mechanism of action. Fluorescence spectroscopy measurements were conducted to obtain the CMC values of each compound, which were complemented by QCM-D and EIS measurements to track the concentration-dependent effects of the two compounds on model phospholipid membranes in terms of three-dimensional membrane morphological changes and ionic permeability changes, respectively. Our findings demonstrate that monoundecanoin potently disrupts phospholipid membranes in an irreversible manner whereas undecanoic acid causes reversible membrane disruption, which suggests that monoundecanoin could also be a promising anti-infective agent. Taken together with biophysical data on other fatty acids and monoglycerides, the results further reinforce the importance of micellar aggregation in enabling membrane-disruptive properties and support that precision tuning of antimicrobial lipid properties such as chain length can directly impact the degree of potency and mechanism of action.

## Materials and methods

### Materials

1,2-Dioleoyl-*sn*-glycero-3-phosphocholine (DOPC) was purchased from Avanti Polar Lipids, Inc. (Alabaster, AL, USA). Undecanoic acid and monoundecanoin were acquired from Larodan AB (Stockholm, Sweden). Phosphate-buffered saline (PBS, pH 7.4, Gibco, Carlsbad, CA, USA) was prepared in Milli-Q-treated water (> 18 MΩ·cm) (Millipore, Billerica, MA, USA).

### Preparation of antimicrobial lipid solutions

The appropriate amounts of antimicrobial lipids in powder form were first measured by analytical mass balance and then dissolved in ethanol to prepare stock solutions with an initial concentration of 800 mM. The ethanolic stock solutions were then diluted 100 times to an 8 mM concentration with PBS buffer [pH 7.4]. Prior to the experiment, the samples were heated in a 70 °C water bath for 30 min, cooled down to room temperature, and then further diluted to the desired test concentrations to enhance solubilization.

### Fluorescence spectroscopy

Fluorescence spectroscopy experiments were conducted to determine the CMC values of undecanoic acid and monoundecanoin, using a previously described methodology [37]. All measurements were performed using a Cary Eclipse fluorescence spectrophotometer (Agilent, Santa Clara, CA, USA), with 1-pyrenecarboxaldehyde (1-pyCHO) serving as the fluorescent probe. Methanol was mixed with a 5 mM aliquot of 1-pyCHO in a glass vial to yield a probe solution with a final concentration of 50 mM, after which the solution was allowed to dry for 30 min. Then, the dried probe was hydrated by adding the desired concentration of antimicrobial lipid sample in PBS buffer solution, followed by vortexing. The excitation wavelength was set at 365.5 nm, and the emission spectrum was collected from 400 to 600 nm. For each measurement, the highest-intensity emission spectrum wavelength was recorded and six technical replicates were performed.

### Quartz crystal microbalance-dissipation (QCM-D)

To temporally track how undecanoic acid and monoundecanoin disrupt SLBs, quartz crystal microbalance-dissipation (QCM-D) experiments were carried out using a Q-Sense E4 instrument (Biolin Scientific, Gothenburg, Sweden), as previously described [37]. The mass and viscoelastic characteristics of the SLB adsorbate were determined in terms of tracking the QCM-D resonance frequency (Δ*f*) and energy dissipation (Δ*D*) signals, respectively, for the oscillating sensor chip [44]. The sensor chip (model no. QSX 303, Biolin Scientific) had a 50-nm-thick, sputter-coated silicon dioxide layer and a fundamental frequency of 5 MHz. Prior to each experiment, the sensor chips were successively rinsed with water and ethanol and dried under flowing nitrogen gas. To remove organic contaminants, the sensor surface was oxygen-plasma treated for 1 min using a CUTE-1MPR oxygen plasma equipment (Femto Science Inc., Hwaseong, Republic of Korea) before immediately mounting the sensor chips in the measurement chambers. To establish baseline signals, PBS solution was first injected into each measurement chamber. Afterwards, zwitterionic SLBs composed of 1,2-dioleoyl-*sn*-glycero-3-phosphocholine (DOPC) phospholipid were fabricated by using the solvent-assisted lipid bilayer (SALB) approach [45, 46]. Briefly, after establishing a baseline signal in PBS solution, the bulk solution was first exchanged with isopropanol, then with 0.5 mg/mL DOPC lipid in isopropanol, and lastly with a rapid solvent exchange back to PBS [45, 47]. All liquid samples were injected into the measurement chambers at a flow rate of 50 µL/min by using a peristaltic pump (Reglo Digital, Ismatec, Glattbrugg, Switzerland). Data were collected using the Q-Soft software program (Biolin Scientific AB). Data analyses were conducted using the Q-Tools (Biolin Scientific AB) and OriginPro (OriginLab, Northampton, MA) software programs. The presented QCM-D data were from the fifth overtone.

### Electrochemical impedance spectroscopy (EIS)

To investigate membrane leakage caused by the antimicrobial lipids, swept-frequency electrochemical impedance spectroscopy (EIS) measurements were performed using a tBLM platform, as previously described [48]. Alternating current (AC) impedance spectroscopy was conducted using a 25 mV peak-to-peak AC excitation at a frequency range of 0.1–2000 Hz for all measurements using a tethaPod reader (SDx Tethered Membranes, Sydney, Australia). A gold electrode slide precoated with a benzyl-disulfide ethylene glycol T10 monolayer composed of benzyldisulphide polyethylene glycol phytanyl (tether) and hydroxyl-terminated benzyldisulphide tetra-ethylene glycol (spacer) molecules in a 1:9 molar ratio was supplied in ethanol and stored at − 20 °C. Prior to the experiment, the precoated slide was rinsed with ethanol, partially dried, and assembled in a tethaPlate cartridge (SDx Tethered Membranes) to allow the subsequent fabrication of the tBLM platform using the solvent-exchange technique [48]. Then, 8 µL of a 3 mM ethanolic mobile lipid phase consisting of C20 glycerol diphytanyl ether (DPEPC) lipid and zwitterionic C20 diphytanyl-diether-phosphatidylcholine lipid in a 3:7 molar ratio was introduced into each channel of the six-channel tethaPlate cartridge. Following a 2-min incubation period, each channel was rinsed three times with 100 µL of PBS solution. The tethaPlate cartridge was then plugged into the tethaPod reader to obtain the measurement readouts and to characterize tBLM formation. Conductance (G_m_) and capacitance (C_m_) values of < 1 µS and ~ 1 µF/cm^2^ measured on a 2.1 mm^2^ gold electrode were considered to be the baseline ranges for tBLMs formed in PBS [pH 7.2]. All experiments were performed in triplicate and representative plots are presented. Data processing were carried out using the tethaQuick (SDx Tethered Membranes) and data analyses were performed using the OriginPro (OriginLab, Northampton, MA, USA) software program.

## Results and discussion

### CMC measurements

The critical micelle concentration (CMC) of a surfactant, such as antimicrobial lipids, refers to the concentration at and above which individual molecules self-assemble in aqueous solution to form nanostructures known as micelles [49]. Since the CMC value can vary with environmental factors [50–52], we determined the CMC values of undecanoic acid and monoundecanoin in PBS. The fluorescence emission spectrum of the 1-pyCHO probe was measured in aqueous solutions containing different concentrations of either compound. When 1-pyCHO molecules are located in the hydrophobic interior of micelles, there is a drop in the maximum-intensity emission wavelength and the lowest compound concentration at which a drop occurs is defined as the CMC value [35, 52, 53].

**Fig. 2:**
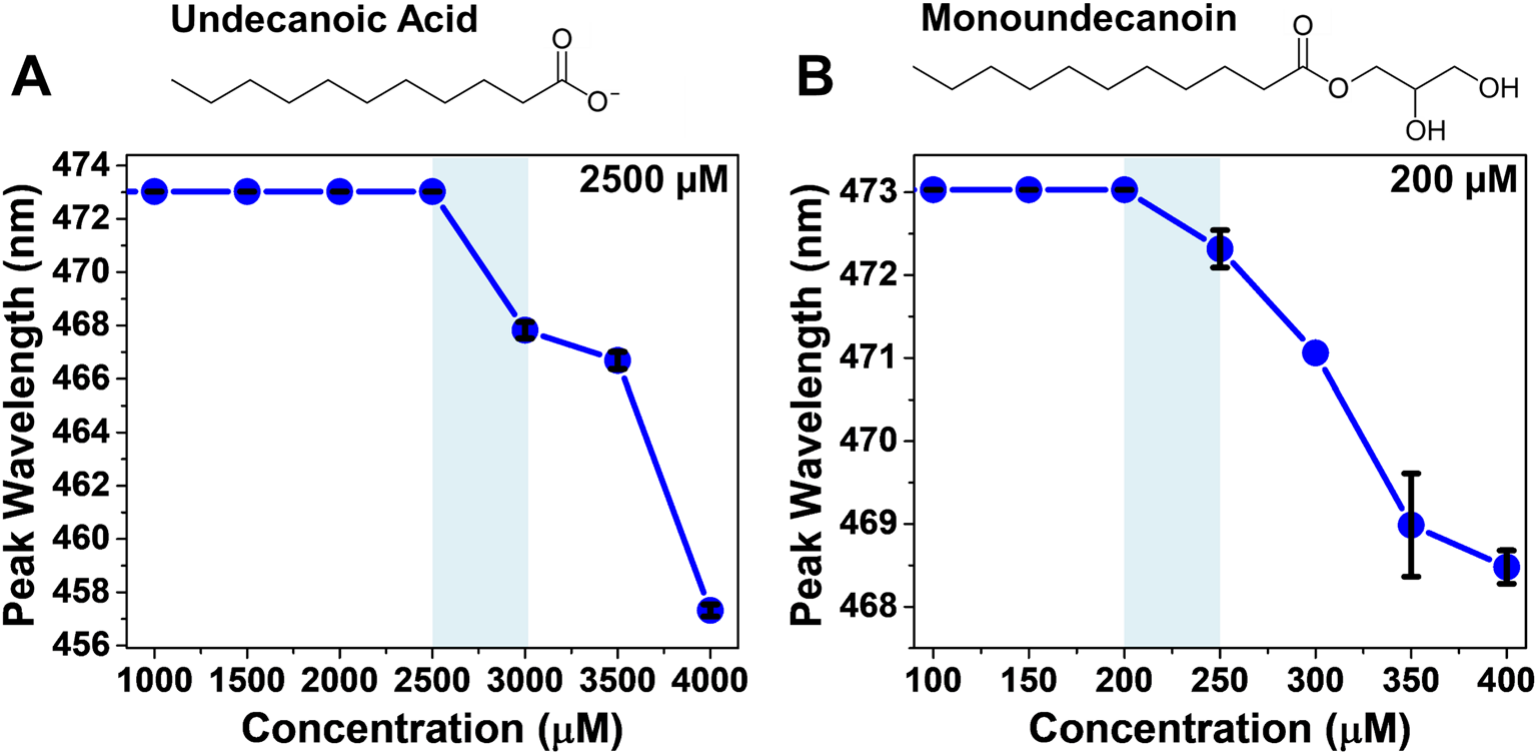
Determination of CMC values for undecanoic acid and monoundecanoin. Fluorescence emission peak wavelength as a function of compound concentrations in PBS solution are presented for (**A**) undecanoic acid and (**B**) monoundecanoin. Each data point reflects the mean of six technical replicates as well as the standard deviation (expressed as error bars). The shaded region highlights the CMC value for the compounds, which is the highest concentration at which no wavelength shift occurs. The molecular structure of each compound is shown above

Figure 2 presents the peak wavelength of the fluorescence emission spectrum as a function of compound concentration. In neat PBS, the peak wavelength of the 1-pyCHO probe in the fluorescence emission spectrum was 473 nm, which agreed with literature values [54]. As the compound concentration increased, there was eventually a drop in the peak wavelength for each compound. The CMC values of undecanoic acid and monoundecanoin in PBS were 2500 µM and 200 μM, respectively. The lower CMC value of monoundecanoin can be attributed to its nonionic character, which leads to a greater tendency to self-assemble as compared to the negatively charged undecanoic acid [37, 55]. Moreover, these CMC values of undecanoic acid and monoundecanoin are in between the corresponding values of their 10- and 12-carbon long analogues, reinforcing that hydrocarbon tail length directly influences micellar aggregation properties. Based on the measured CMC values, appropriate compound concentrations were selected for further testing in order to investigate concentration-dependent biophysical interactions with SLB platforms.

### QCM-D tracking of membrane-disruptive interactions

The QCM-D technique was employed to track the effects of adding undecanoic acid and monoundecanoin to prefabricated SLBs on silica-coated sensor chips by monitoring changes in the measured resonance frequency (Δ*f*) and energy dissipation (Δ*D*) shifts of the adsorbed lipid layer. The Δ*f* shift reflects changes in the acoustic mass of the adsorbed layer, whereby a negative Δ*f* shift corresponds to increased acoustic mass that can arise from biomolecular mass binding or a membrane morphological change. On the other hand, the Δ*D* shift is more sensitive to viscoelastic properties in the adsorbed lipid layer and a positive Δ*D* shift typically relates to an increase in adlayer viscoelasticity such as more hydrodynamically coupled solvent [56]. Importantly, in the context of studying the membrane-disruptive properties of antimicrobial lipids, the time-resolved Δ*f* and Δ*D* shifts can be viewed collectively as quantitative measurement signatures, which have been previously correlated with qualitative, time-lapse fluorescence microscopy imaging results for various fatty acids and monoglycerides [37, 38], in order to distinguish whether resulting interactions cause mainly budding- or tubule-like membrane morphological changes based on the QCM-D measurement responses. Hence, QCM-D measurement data can provide a quantitative basis to compare the membrane morphological changes induced by different antimicrobial lipids.

**Fig. 3:**
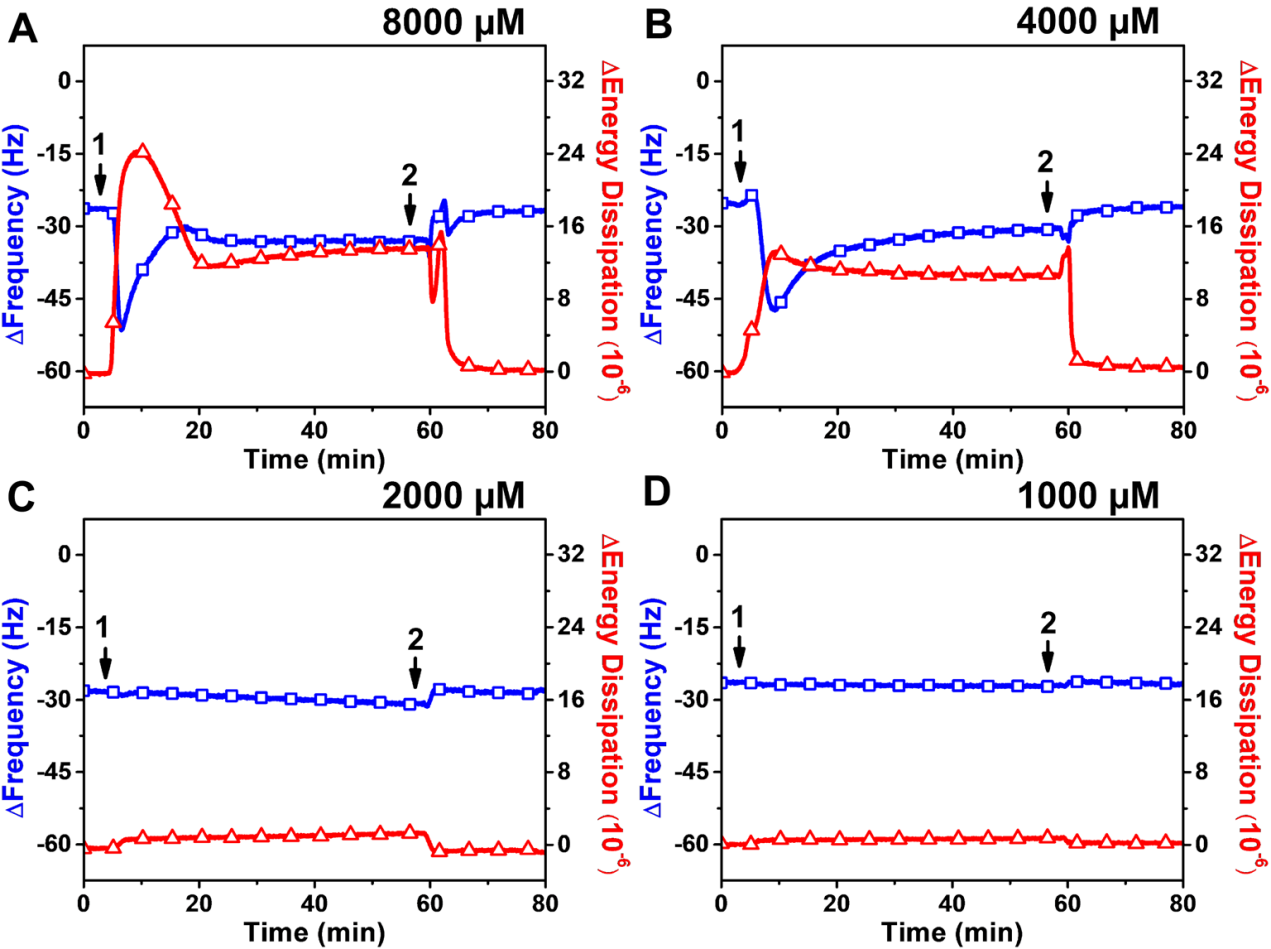
QCM-D tracking of undecanoic acid addition to supported lipid bilayer platform. Time-resolved Δ*f* (blue line with squares) and Δ*D* (red line with triangles) shifts are presented for treating the SLB with (**A**) 8000 µM, (**B**) 4000 µM, (**C**) 2000 µM, and (**D**) 1000 µM undecanoic acid. The initial baseline measurements were consistent with the typical QCM-D values for supported lipid bilayer formation on a silica surface. After the baseline signals were stabilized for 5 min, undecanoic acid was continually added (arrow 1) for around 50 min before a buffer washing step was performed (arrow 2)

**Fig. 4:**
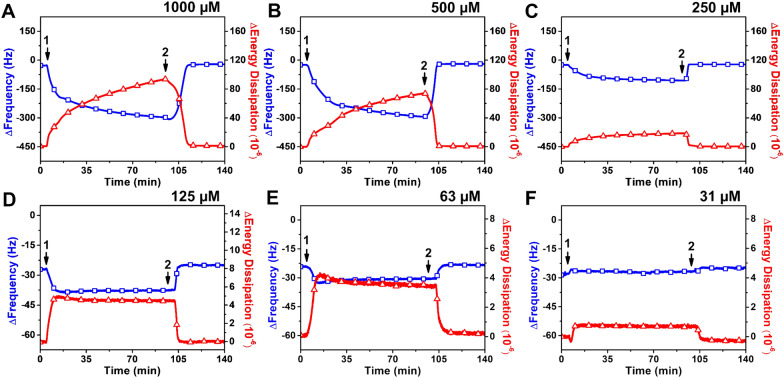
QCM-D tracking of monoundecanoin addition to supported lipid bilayer platform. Time-resolved Δ*f* (blue line with squares) and Δ*D* (red line with triangles) shifts are presented for treating the SLB with (**A**) 1000 µM, (**B**) 500 µM, (**C**) 250 µM, (**D**) 125 µM, (**E**) 63 µM, and (**F**) 31 µM monoundecanoin. The initial baseline measurements were consistent with the typical QCM-D values for supported lipid bilayer formation on a silica surface. After the baseline signals were stabilized for 5 min, monoundecanoin was continually added (arrow 1) for around 90 min before a buffer washing step was performed (arrow 2)

In our experiments, zwitterionic SLB was first formed on the sensor chip by using the solvent-assisted lipid bilayer (SALB) formation method. The corresponding Δ*f* and Δ*D* shifts for the SLB formation step were − 26 ± 2 Hz and 0.3 ± 0.2 × 10^− 6^, which indicate successful fabrication [45]. Afterwards, the measurement signals were stabilized following a buffer washing step and then various test concentrations of undecanoic acid or monoundecanoin were added to the SLB under continuous flow conditions. The time-resolved QCM-D data are presented in Figs. 3 and 4. Note that the normalized Δ*f* and Δ*D* shifts at the initial time point correspond to the values for a prefabricated SLB on the silica-coated sensor chip surface, which were reset to focus on the interaction kinetics due to antimicrobial lipid addition.

#### Undecanoic acid

Figure 3 presents the QCM-D measurement responses corresponding to the addition of different undecanoic acid concentrations to the SLB platform. For 8000 µM undecanoic acid, there was a rapid decrease in Δ*f* and increase in Δ*D* to −51 Hz and 24 × 10^−6^, respectively (Fig. 3A). After reaching inflection points, the Δ*f* shift increased to about −34 Hz while the Δ*D* shift decreased to and stabilized at around 13 × 10^−6^. The subsequent increase in the Δ*f* signal suggests that undecanoic acid caused membrane destabilization, which is also consistent with the large increase in the Δ*D* signal that typically arises from membrane shape transformations. A buffer washing step led to Δ*f* and Δ*D* shifts reaching around −26 Hz and 1 × 10^−6^ respectively. Similarly, 4000 µM undecanoic acid saw a simultaneous decrease in Δ*f* and increase in Δ*D* to −47 Hz and 13 × 10^−6^ respectively. The Δ*f* shift then returned upward to about − 30 Hz while the Δ*D* shift decreased to 11 × 10^−6^. A washing step caused the Δ*f* and Δ*D* shifts to return to about −26 Hz and 1 × 10^−6^ respectively (Fig. 3B). These final values indicate that the SLB adlayer largely remained on the sensor surface, demonstrating that undecanoic acid induced membrane morphological changes but did not cause appreciable membrane solubilization. Rather, the pattern of the Δ*f* and Δ*D* shifts is characteristic of tubule formation, which has previously been reported for 10- and 12-carbon long, saturated fatty acids [38, 57].

On the other hand, the addition of 2000 µM and 1000 µM undecanoic acid concentrations caused negligible changes in the QCM-D measurement responses, with resulting Δ*f* and Δ*D* shifts of less than −2 Hz and 2 × 10^−6^ respectively (Fig. 3C, D). These values indicate that undecanoic acid mainly exhibits membrane-disruptive activity at concentrations above its CMC value (2500 µM), while lower concentrations in which undecanoic acid is present in the monomeric state did not cause appreciable membrane disruption.

#### Monoundecanoin

Figure 4 presents the QCM-D measurement responses corresponding to the addition of different monoundecanoin concentrations to the SLB platform. For 1000 µM monoundecanoin, there was a simultaneous decrease in the Δ*f* signal and increase in the Δ*D* signal to −298 Hz and 94 × 10^−6^, respectively (Fig. 4A). After buffer washing, the Δ*f* and Δ*D* signals reached around −28 Hz and 3 × 10^−6^, respectively. The pattern of measurement responses is consistent with extensive membrane budding [35, 57], while the final responses indicate that some degree of membrane disruption persisted. Upon treatment with 500 µM monoundecanoin, there was a similar trend in measurement responses, while the Δ*f* and Δ*D* shifts were smaller and around − 295 Hz and 74 × 10^−6^, respectively (Fig. 4B). In that case, a subsequent buffer washing step led to an increase in the Δ*f* signal and a decrease in the Δ*D* signal to around − 25 Hz and 3 × 10^−6^, respectively. Treatment with 250 µM monoundecanoin also caused membrane disruption to a lesser extent, and the corresponding Δ*f* and Δ*D* shifts were around −106 Hz and 19 × 10^−6^, respectively (Fig. 4C). After a buffer washing step, the final Δ*f* and Δ*D* shifts were around − 30 Hz and 3 × 10^−6^, respectively.

In marked contrast, treatment with 125 or 63 µM monoundecanoin concentrations led to appreciably smaller measurement responses, with Δ*f* and Δ*D* shifts of less than −15 Hz and 5 ⋅ 10^−6^, respectively (Fig. 4D, E). In these cases, the final Δf and ΔD values were equivalent to the baseline values, indicating minimal membrane disruption. Moreover, for 31 µM monoundecanoin, there were negligible QCM-D measurement responses, indicating that monoundecanoin was inactive at that concentration (Fig. 4F). Together, these data support that monoundecanoin mainly exhibits membrane-disruptive behavior at ≥ 250 µM, which is consistent with its CMC value of 200 µM.

### EIS analysis of membrane-disruptive mechanisms

To further characterize the mechanisms by which undecanoic acid and monoundecanoin disrupt phospholipid membranes, electrochemical impedance spectroscopy (EIS) measurements were conducted using a tethered bilayer lipid membrane (tBLM) platform that is separated from a gold electrode surface by a nm-scale ionic reservoir. The EIS technique tracks time-resolved changes in the conductance (G_m_) and capacitance (C_m_) signals corresponding to the electrochemical properties of the tBLM platform, which are related to ionic leakage through the tethered lipid bilayer and electrical charge stored within the lipid bilayer, respectively.

When membrane disruption occurs, there is often a decrease in lipid packing within the tBLM that causes a G_m_ shift increase. Membrane thinning events can also cause a C_m_ shift decrease. In addition to time-resolved G_m_ and C_m_ signals, the EIS data can also be presented using a Bode plot format that depicts the phase angle as a function of frequency, which is particularly useful for comparing measurement responses before and after compound treatment. Following this approach, the test compound concentrations for the EIS measurements were chosen to be two times higher and lower than their corresponding CMC values. After establishing the tBLM baseline signals, the test compounds were added and incubated for 30 min, followed by a PBS buffer washing step.

#### Undecanoic acid

**Fig. 5:**
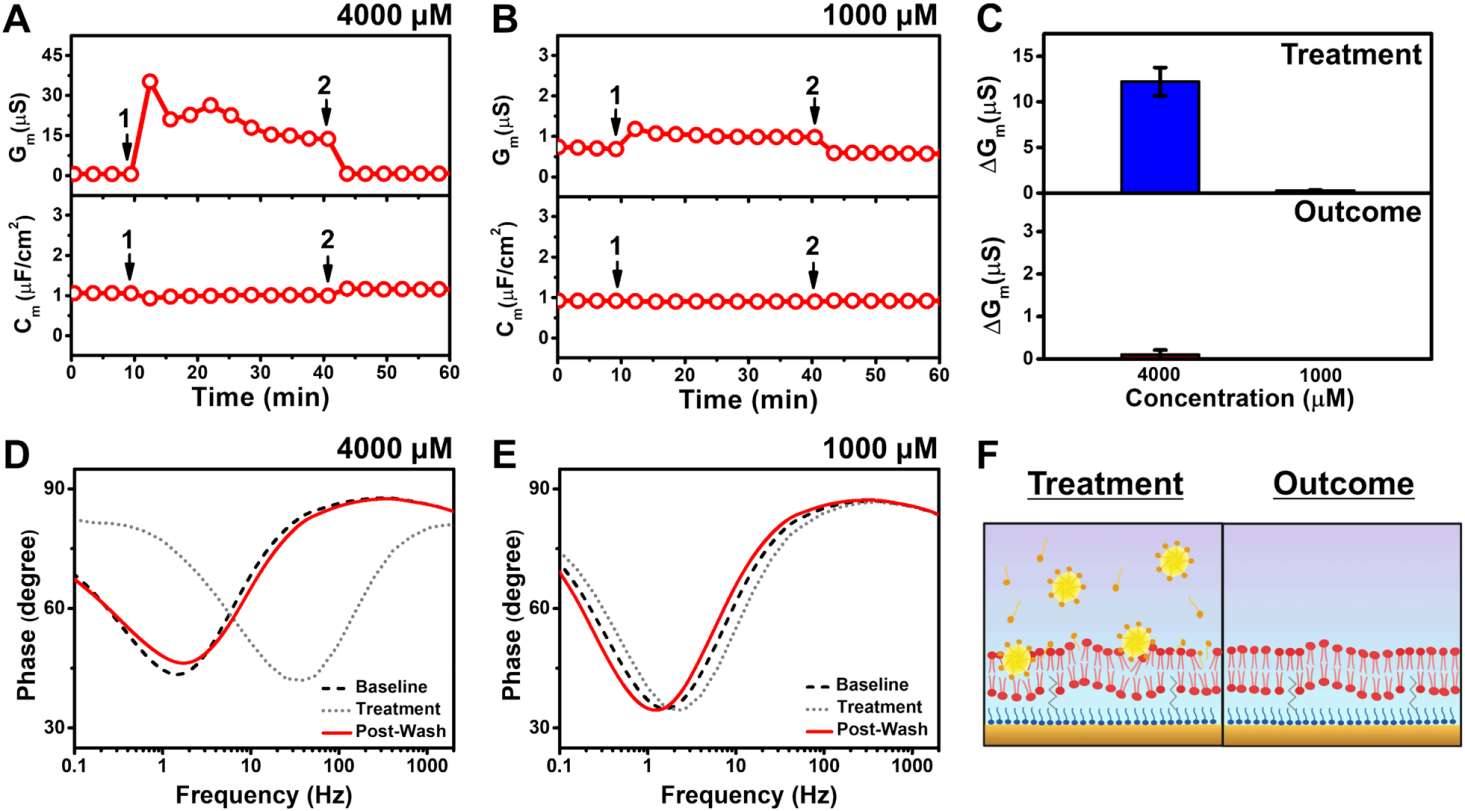
EIS investigation of undecanoic acid addition to the tBLM platform. Time-resolve changes in conductance (G_m_) and capacitance (C_m_) signals following the addition of (**A**) 4000 µM and (**B**) 1000 µM undecanoic acid to the tBLM platform. The initial baseline measurements correspond to the fabricated tBLM platform in PBS solution on the gold electrode. After 10 min of establishing the baseline signals, undecanoic acid was added (arrow 1) and incubated for 30 min before buffer washing was performed (arrow 2). (**C**) Summary of G_m_ shifts upon compound addition (top panel) and after buffer washing (bottom panel) relative to the tBLM baselines. Bode plots describing the phase shifts of the system from baseline signals in response to treatment with (**D**) 4000 µM or (**E**) 1000 µM undecanoic acid and buffer washing. (**F**) Schematic illustration based on the EIS data of how 4000 µM undecanoic acid interacts with the tBLM and the resulting tBLM after undecanoic acid was removed by buffer washing

Figure 5 presents the time-resolved EIS results following tBLM treatment with 4000 µM and 1000 µM undecanoic acid. For 4000 µM undecanoic acid, the G_m_ signal increased up to a transient peak value of around 35 µS and stabilized at around 14 µS (Fig. 5A). A minimal shift in the C_m_ signal to ~ 1 µF/cm^2^ was also observed. Upon buffer washing, the G_m_ and C_m_ signals returned to nearly the corresponding baseline values of around 0.7 µS and 1.1 µF/cm^2^, respectively

In the case of 1000 µM undecanoic acid, the G_m_ signal increased slightly to around 1 µS and no C_m_ shift was observed (Fig. 5B). Similar to the previous case, the G_m_ and C_m_ signals were recovered upon buffer washing. The corresponding G_m_ shifts upon treatment with 4000 µM and 1000 µM undecanoic acid and after buffer washing relative to the baseline signals are summarized in Fig. 5C. 4000 µM and 1000 µM undecanoic acid induced G_m_ shifts of 12 ± 2 µS and 0.3 ± 0 µS, respectively, and the final shifts were reduced to 0.1 ± 0.1 µS and around − 0.1 µS, respectively, upon washing. The Bode plot for the case of 4000 µM undecanoic acid shows that the phase minima shifted to a higher frequency upon treatment; however, after buffer washing, the final value was similar to the baseline level (Fig. 5D). On the other hand, the addition of 1000 µM undecanoic acid to the tBLM platform caused only slight changes to the plot features (Fig. 5E). These trends indicate concentration-dependent membrane disruption, which is consistent with CMC-dependent activity, and support that undecanoic acid causes reversible membrane disruption.

Based on the information obtained from the EIS results, the proposed mechanism of action of 4000 µM undecanoic acid interacting with phospholipid membranes is illustrated in Fig. 5F. Above CMC, in this case, undecanoic acid is mainly present as micelles. Upon the addition of undecanoic acid to the measurement chamber containing the tBLM, the micelles adsorb onto the tBLM surface, partially insert into the lipid bilayer, and affect lipid packing, which thereby lowers the surface tension and increases ionic permeability [58]. As the membrane insertion of undecanoic acid is relatively weak considering its short alkyl chain length and it has low inter-leaflet translocation ability due to its negatively charged headgroup, it is reasonable that undecanoic acid was rinsed away upon the buffer washing step. The disrupted lipid packing was then recovered due to hydrophobic interactions between phospholipid molecules, resulting in recovery of the intact tBLM.

#### Monoundecanoin

**Fig. 6:**
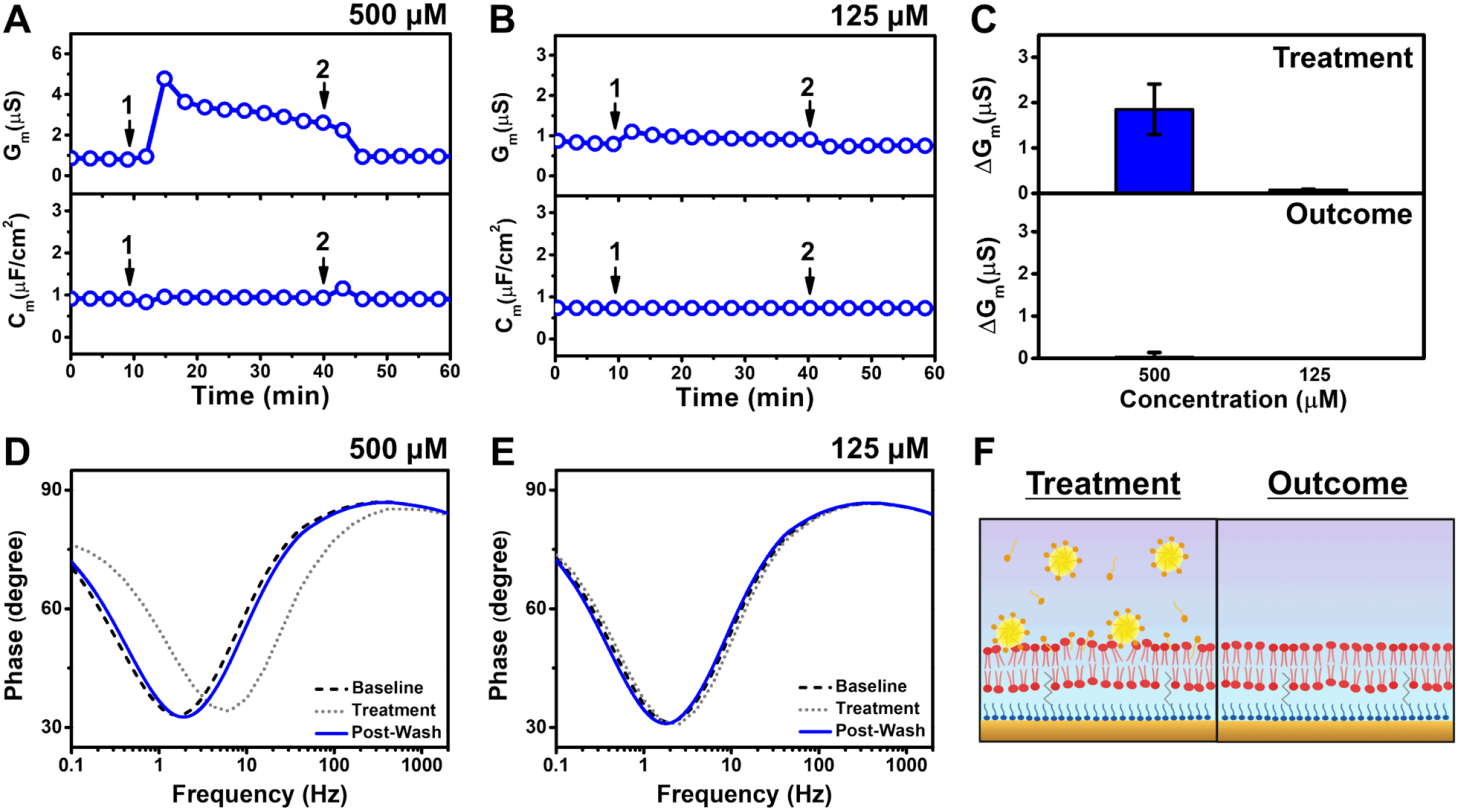
EIS investigation of monoundecanoin addition to the tBLM platform. Time-resolve changes in conductance (G_m_) and capacitance (C_m_) signals following the addition of (**A**) 500 µM and (**B**) 125 µM monoundecanoin to the tBLM platform. The initial baseline measurements correspond to standard values indicating formation of the tBLM platform in PBS solution on the gold electrode. After 10 min of establishing the baseline, monoundecanoin was added (arrow 1) and incubated for 30 min before buffer washing was performed (arrow 2). **C** Summary of G_m_ shifts upon compound addition (top panel) and after buffer washing (bottom panel) relative to the tBLM baselines. Bode plots describing the phase shifts of the system from baseline signals in response to treatment with (**D**) 500 µM or (**E**) 125 µM monoundecanoin and buffer washing. **F** Schematic illustration based on the EIS data of how 500 µM monoundecanoin interacts with the tBLM and the resulting tBLM after monoundecanoin was removed by buffer washing

Figure 6 presents the time-resolved EIS results following tBLM treatment with 500 µM and 125 µM monoundecanoin. Upon 500 µM monoundecanoin addition, the peak G_m_ value was around 4.8 µS and then stabilized at around 2.2 µS (Fig. 6A). After buffer washing, the G_m_ signal recovered to nearly its baseline value of around 0.9 µS. A minimal C_m_ shift was also observed. On the other hand, a minor increase in the G_m_ signal to around ~ 1 µS was observed upon 125 µM monoundecanoin addition, and similarly, the G_m_ signal returned to its baseline value after buffer washing (Fig. 6B). There were no changes in the C_m_ signal in that case. Figure 6C presents a summary of the G_m_ shifts upon treating the tBLM with 500 µM and 125 µM monoundecanoin and after buffer washing. Specifically, the G_m_ shifts upon 500 µM and 125 µM monoundecanoin treatment were 1.9 ± 0.6 µS and 0.08 ± 0.01 µS, respectively. After a buffer washing step, both signals recovered to reach G_m_ shifts of around 0.2 µS and − 0.05 µS, respectively. The Bode plots further showed a modest increase in the phase minima that corresponded to a higher frequency upon treatment with 500 µM monoundecanoin and the shift returned to the baseline level after buffer washing (Fig. 6D). By contrast, there was no shift seen in the Bode plot upon 125 µM monoundecanoin addition (Fig. 6E). Taken together, these findings support that only 500 µM monoundecanoin induced membrane disruption and the membrane-disruptive effect in this concentration range was reversible, as indicated by baseline signal recovery after the buffer washing step.

The membrane-disruptive effects of the 500 µM monoundecanoin treatment case are schematically presented in Fig. 6F. Following treatment, monoundecanoin micelles adhered to the tBLM and disturbed the packing of lipid molecules within the tethered bilayer, thereby increasing ionic permeability across the membrane. At this relatively low concentration, the interacting monoundecanoin molecules were readily removed during the subsequent buffer washing step, which resulted in recovery of the tBLM platform with electrochemical sealing. Based on these EIS results, when comparing the membrane-disruptive properties of undecanoic acid and monoundecanoin at two-times above their corresponding CMC values, the data support that undecanoic acid causes greater membrane disruption than monoundecanoin on account of larger G_m_ shifts.

**Fig. 7:**
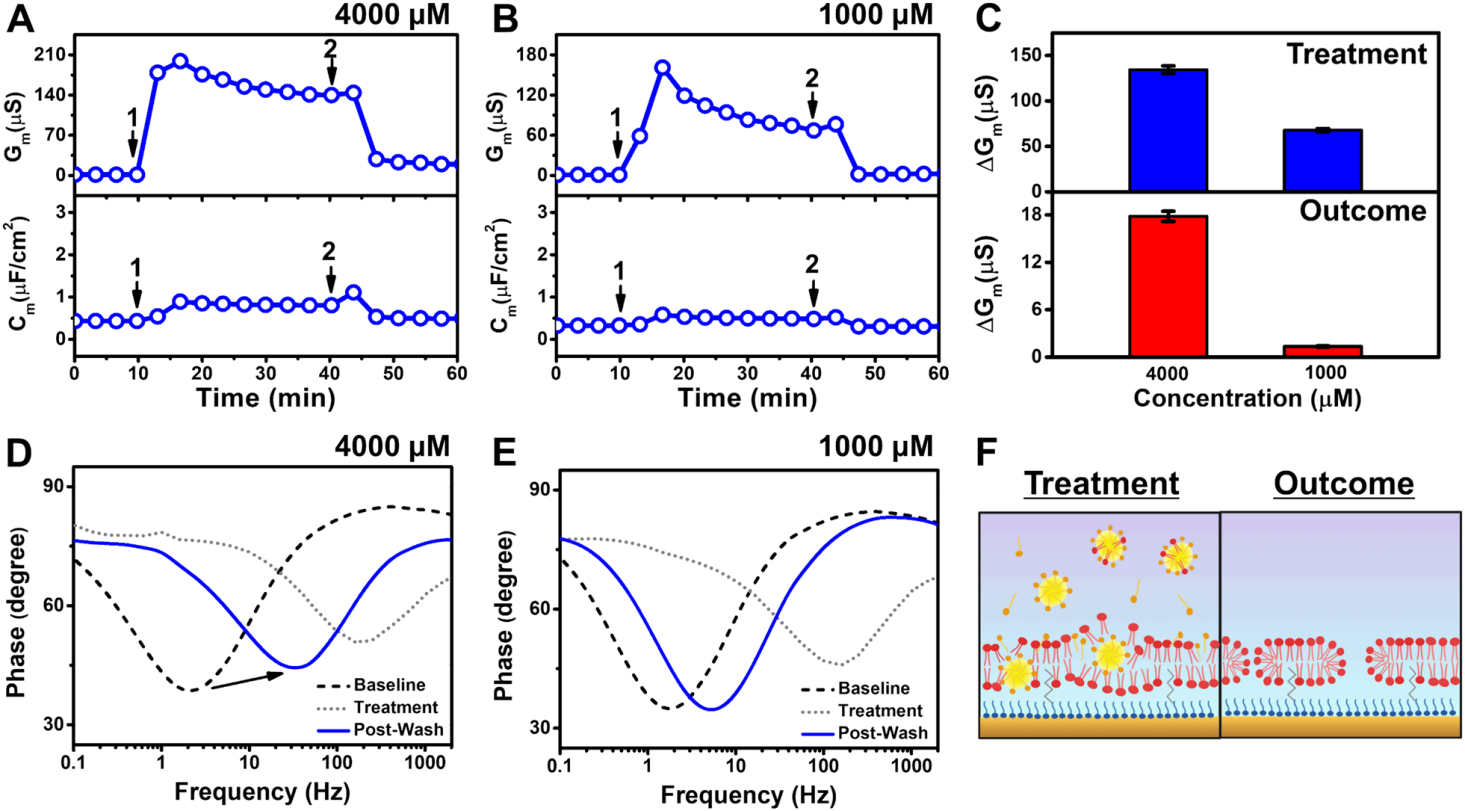
EIS investigation of adding higher monoundecanoin concentrations to the tBLM platform. Time-resolve changes in conductance (G_m_) and capacitance (C_m_) signals following the addition of (**A**) 4000 µM and (**B**) 1000 µM monoundecanoin to the tBLM platform. The initial baseline measurements correspond to the fabricated tBLM platform in PBS solution on the gold electrode. After 10 min of establishing the baseline signals, monoundecanoin was added (arrow 1) and incubated for 30 min before buffer washing was performed (arrow 2). **C** Summary of G_m_ shifts upon compound addition (top panel) and after buffer washing (bottom panel) relative to the tBLM baselines. Bode plots describing the phase shifts of the system from baseline signals in response to treatment with (**D**) 4000 µM or (**E**) 1000 µM monoundecanoin and buffer washing. **F** Schematic illustration based on the EIS data of how 4000 µM monoundecanoin interacts with the tBLM and the resulting tBLM after monoundecanoin was removed by buffer washing

To have a direct comparison in terms of molar units, we also tested 4000 µM and 1000 µM monoundecanoin, which were 20 × and 5 × greater concentrations than its CMC and matched the tested concentrations of undecanoic acid (Fig. 7). In the case of 4000 µM monoundecanoin, there was a rapid and appreciable G_m_ shift increase to a peak value of around 200 µS along with a C_m_ increase to around 0.9 µF/cm^2^ (Fig. 7A). The G_m_ signal then gradually stabilized at around 140 µS. After buffer washing, the G_m_ and C_m_ values decreased to around 22 µS and 0.5 µF/cm^2^, respectively, but did not return to baseline values. On the other hand, upon 1000 µM monoundecanoin addition, the peak G_m_ value reached around 160 µS, followed by the signal stabilization at around 74 µS. Simultaneously, the C_m_ value increased to 0.5 µF/cm^2^. Similar to the aforementioned case, upon buffer washing, the G_m_ and C_m_ signals decreased to around 1.3 µS and 2.2 µF/cm^2^, respectively (Fig. 7B). Comparatively, the G_m_ shifts corresponding to 4000 µM and 1000 µM monoundecanoin addition were around 135 ± 4 µS and 68 ± 2 µS, respectively (Fig. 7C). Following buffer washing, the G_m_ shifts decreased to around 18 ± 1 µS and ~ 1.3 µS, respectively, but did not return to the baseline values due to irreversible membrane disruption. The Bode plots verified that treatment with 4000 µM and 1000 µM monoundecanoin resulted in irreversible membrane disruption, with 4000 µM monoundecanoin inducing greater disruption (Fig. 7D, E).

Based on the EIS results, the membrane-disruptive effects of 4000 µM monoundecanoin are schematically illustrated in Fig. 7F. At this high concentration, there is an appreciably higher micelle concentration in the bulk solution and their interactions with the phospholipid membrane caused more extensive membrane disruption, which resulted in irreversible damage. While the CMC is an important concept associated with conferring membrane-disruptive activity of antimicrobial lipids, this finding supports that increasing the antimicrobial lipid concentration, in this case that of monoundecanoin, can enhance the degree of membrane-disruptive activity. In particular, treatment with a sufficiently high monoundecanoin concentration caused irreversible membrane disruption, whereas lower but still active concentrations above the CMC only caused reversible damage. This might be rationalized by taking into consideration the degree to which membrane remodeling can relieve strain-induced antimicrobial lipid interactions. As such, at equivalent concentrations, monoundecanoin caused greater membrane disruption than undecanoic acid, which provides an additional reason to support why monoglycerides are typically more potent and active than their corresponding fatty acids in addition to the difference in CMC values.

Overall, the EIS results demonstrated that undecanoic acid and monoundecanoin triggered reversible membrane disruption at their respective 2× CMC values while higher concentrations of monoundecanoin induced irreversible membrane disruption. In terms of benchmarking performance compared to other biologically active antimicrobial lipids, we may also add that 11-carbon undecanoic acid induced appreciably greater membrane disruption at 2 × CMC, as compared to 12-carbon lauric acid [48]. Furthermore, monoundecanoin also induced moderately greater membrane disruption than GML at 2 × CMC [48]. Considering that shorter-chain antimicrobial lipids typically have greater solubility in aqueous environments as well, these findings support that undecanoic acid and monoundecanoin are attractive candidates for further development as membrane-disrupting antimicrobial lipids. Note that undecanoic acid is already used within antifungal creams while our results suggest that it might also be useful for other aqueous-based formulations such as lipid-based nanoparticles [59].

## Conclusion

In this study, we have utilized biophysical measurement strategies to investigate the membrane-disruptive properties of one of the most biologically active odd-chain fatty acids, 11-carbon undecanoic acid, and its monoglyceride derivative, monoundecanoin. While even-chain fatty acids and monoglyceride derivatives have been increasingly studied using biophysical approaches, their odd-chain counterparts have been strictly evaluated in terms of microbial inhibition. Our findings establish that undecanoic acid and monoundecanoin disrupt phospholipid membranes in a CMC-dependent manner and QCM-D experiments showed that they cause membrane tubulation and budding in SLB platforms, respectively. Using a tBLM platform, EIS testing further revealed that monoundecanoin caused irreversible membrane disruption at sufficiently high concentrations whereas undecanoic acid induced reversible membrane disruption. Notably, the observed degrees of membrane-disruptive potency of undecanoic acid and monoundecanoin fall in line with current mechanistic understanding about how 10- and 12-carbon long antimicrobial lipids disrupt phospholipid membranes in terms of antimicrobial lipid concentration, i.e., a minimum concentration is required for extensive disruption. This trend reinforces that micellar aggregation plays an important role in contributing to the membrane-disruptive activity of antimicrobial lipids while, in the case of monoundecanoin, we observed that increasing the compound concentration well beyond the CMC can induce irreversible membrane disruption as opposed to reversible disruption at lower, still active concentrations closer to the CMC. Together, these findings establish that antimicrobial lipid concentration is an important determinant of membrane-disruptive activity not only in terms of CMC-related properties but also in terms of modulating the degree of membrane disruption. Additionally, our findings demonstrate how nanoscience tools such as the QCM-D and EIS techniques can be applied in tandem to characterize the mechanism of action and potency of antimicrobial lipid candidates, in this case identifying how 11-carbon undecanoic acid and monoundecanoin exhibit high levels of membrane-disruptive activity compared to their more conventionally used, 12-carbon analogues. In future work, it will also be illuminating to utilize solution-phase biophysical techniques such as small-angle X-ray scattering (SAXS) in order to further investigate how the molecular properties of antimicrobial lipids affect micellar shape and packing, especially since past findings on related detergents have reported ellipsoidal micelle shapes and that the aggregation number tends to increase with longer alkyl chains [60]. Aside from modulating the nanoarchitecture properties of the model membrane platform itself, such insights might pave the way towards rationally tuning the molecular-level properties of antimicrobial lipids in different nanoarchitectures and provide control over membrane-disruptive properties.

## Data Availability

The data presented in this study are available upon reasonable request from the corresponding authors.

## Abbreviations

CMC: Critical micelle concentration
DOPC: 1,2-Dioleoyl-*sn*-glycerol-3-phosphocholine
EIS: Electrochemical impedance spectroscopy
GML: Glycerol monolaurate
LA: Lauric acid
LSPR: Localized surface plasmon resonance
PBS: Phosphate-buffered saline
QCM-D: Quartz crystal microbalance-dissipation
SDS: Sodium dodecyl sulfate
SLB: Supported lipid bilayer
tBLM: Tethered lipid bilayer membranes
1-pyCHO: 1-pyrenecarboxaldehyde
